# Immuno markers in newly diagnosed glioblastoma patients underwent Stupp protocol after neurosurgery: a retrospective series

**DOI:** 10.1007/s11060-023-04357-9

**Published:** 2023-08-16

**Authors:** Lorena Gurrieri, Laura Mercatali, Toni Ibrahim, Valentina Fausti, Monia Dall’Agata, Nada Riva, Nicoletta Ranallo, Giuseppe Pasini, Marcella Tazzari, Flavia Foca, Daniela Bartolini, Luca Riccioni, Chiara Cavatorta, Federico Paolo Morigi, Jenny Bulgarelli, Claudia Cocchi, Virginia Ghini, Luigino Tosatto, Giovanni Martinelli, Andrea Pession, Laura Ridolfi

**Affiliations:** 1 Clinical and Experimental Oncology, Immunotherapy, Rare Cancers and Biological Resource Center, IRCCS Istituto Romagnolo per lo Studio dei Tumori (IRST) “Dino Amadori”, Via P. Maroncelli 40, 47014 Meldola, Italy; 2 Preclinic and Osteoncology Unit, Bioscience Laboratory, IRCCS Istituto Romagnolo per lo Studio dei Tumori (IRST) “Dino Amadori”, 47014 Meldola, Italy; 3 Osteoncology, Bone and Soft Tissue Sarcomas, and Innovative Therapies, IRCCS Istituto Ortopedico Rizzoli, 40138 Bologna, Italy; 4 Unit of Biostatistics and Clinical Trials, IRCCS Istituto Romagnolo per lo Studio dei Tumori (IRST) “Dino Amadori”, 47014 Meldola, Italy; 5 Department of Medical Oncology, “Infermi” Hospital, 47921 Rimini, Italy; 6 Pathology Unit, “Maurizio Bufalini” Hospital, 47521 Cesena, Italy; 7 Neurosurgery, “Maurizio Bufalini” Hospital, 47521 Cesena, Italy; 8 Scientific Direcrorate, IRCCS Istituto Romagnolo per lo Studio dei Tumori (IRST) “Dino Amadori”, 47014 Meldola, Italy; 9 Division of Pediatrics, IRCCS Azienda Ospedaliero-Universitaria Di Bologna, 40138 Bologna, Italy

**Keywords:** Glioblastoma, Tumor microenvironment, Temozolomide, Inflammatory markers, Immunotherapy, Immuno-markers

## Abstract

**Purpose:**

The aims of our retrospective study investigated the role of immune system in glioblastoma (GBM), which is the most aggressive primary brain tumor in adults characterized by a poor prognosis. The recurrence rate remains high, probably due to “immune-desert” tumor microenvironment (TME) making GBM hidden from the anti-tumoral immune clearance. Considering this, we aimed to create a panel of prognostic markers from blood and tumor tissue correlating with overall survival (OS) and progression-free survival (PFS).

**Methods:**

Firstly, we analyzed the inflammatory markers NLR and PLR as the ratio of the absolute neutrophil count and absolute platelet count by the absolute lymphocyte count respectively, collected at different time points in the peripheral blood of 95 patients. Furthermore, in 31 patients of the same cohort, we analyzed the formalin-fixed paraffin embedded samples to further compare the impact of circulating and inflammatory markers within the TME.

**Results:**

Patients aged < 60 years and with methylated MGMT showed better OS. While, pre-chemotherapy Systemic Inflammatory Index (SII) < 480 was related to a better OS and PFS, we observed that only CD68+macrophage and CD66b+neutrophils expressed in vascular/perivascular area (V) showed a statistically significant prognostic role in median OS and PFS.

**Conclusions:**

Thus, we underscored a role of SII as predictive value of response to STUPP protocol. Regarding the TME-related markers, we suggested to take into consideration for future studies with new immunotherapy combinations, each component relating to expression of immune infiltrating subsets.

**Supplementary Information:**

The online version contains supplementary material available at 10.1007/s11060-023-04357-9.

## Introduction

Glioblastoma (GBM) is the most lethal type of glioma with a median survival time of ∼14 months despite surgical resection, concomitant radiotherapy and chemotherapy following by chemotherapy as Stupp regimen [1, 2]. The O6-methylguanine-DNA methyltransferase (MGMT) promoter methylation status as a predictive factor for the response to temozolomide is well established [3–5]. In the new 2021 WHO Classification, molecular biomarkers such as isocitrate dehydrogenase 1 (IDH1) and MGMT had confirmed their prognostic role [6]. Unfortunately, most GBM are IDH-1 wild type and unmethylated MGMT becomig aggressive and resistant to therapies included the new immune checkpoint inhibitors due to the “cold tumor” phenotype [7–9]. Moreover, GBM patients are poor for predictive serum markers. Emerging studies highlighted the crucial role of inflammatory process in pathogenesis of many solid cancers [10–14]. Generally, the inflammatory response is characterized by changes in neutrophil/lymphocyte ratio (NLR) and platelet/lymphocyte ratio (PLR) [15–17]. In 2017, Kaya et al., defined a systemic inflammatory response (SIR) both on the individually NLR and PLR and in combination considering the presence of a SIR if NLR ≥ 5 or PLR ≥ 150 [18]. In addition,the heterogeneous TME could be responsible for the drug’s failure [19, 20]. Therefore, additional marker to predict the outcome of GBM are urgent and necessary. Microglia has the greatest load of immune cells ready to defend the brain against pathogens [21–26]. Weenink et al., explained how T cells could extravasate from blood vessels in a brain cancer. However, the intratumoral CD8+T cells in GBM is usually small (0–12%) when compared to extracranial tumor types [27]. CD8+ can be related to a favorable survival in GBM [28]. In addition to T cells, tumor-associated macrophages (TAMs) are highly present in GBM, and represent up to 50% of intratumoral immune cells involving in CD8+ suppression. TAMs are able to promote tumor growth, correlate with tumor grade and progression [29]. Comparing to regulatory T (Treg) cells, TAMs are a strong predictor of survival in GBM [30]. In 2018, Orrego et al., investigated the association between the density of CD3+, CD4+, CD8+, CD20+ tumor-infiltrating lymphocytes (TILs), monocytes, CD68+ and CD163+ macrophages with MGMT methylation status in relation to prognosis. On 43 GBM, they observed a low number of CD3+in larger tumor size, a low CD4+ in methylated MGMT and a low CD8+ related to methylation [31]. Finally, a lower number of CD4+ and CD8+ TILs seems to be associated with better outcome. The aims of our retrospective study were to analyze the prognostic and predictive role of systemic inflammatory markers, to evaluate the TME compositions and to create a tool of potential prognostic and predictive circulating and tissue markers.

## Materials and methods

### Patients and data collection

This retrospective multicentric study included a case series of patients with a histological diagnosis of GBM referred to the Rare Tumors Center (IRCCS IRST-Meldola) and Oncology Unit of Rimini between January 2008 and 2019. All patients underwent neurosurgery and 6 weeks of radiation for 60 Gy plus concomitant daily temozolomide (75 mg/m2/day, 7 days/week from the first to the last day of radiotherapy), followed by adjuvant temozolomide (150–200 mg/m2/day for 5 days during each 28-day cycle). The patients were excluded if they had only biopsy or they received the sequential treatment (radiotherapy then temozolomide) or only chemo. The patients were treated with steroid therapy pre and post surgery. We recorded clinical and molecular data about MGMT methylation status, type of surgery, tumor site, symptoms and radio-chemotherapy. NLR and PLR were computed as the ratio of the absolute neutrophil count and absolute platelet count by the absolute lymphocyte count respectively. Systemic Inflammatory Index (SII) was calculated as platelet × neutrophil/lymphocyte count. The blood markers were evaluated: before neurosurgery, before radio-chemotherapy and at the end of Stupp regimen. Friedman’s test and Bonferroni post-hoc comparison were used to test the differences over time. Time-dependent receiver operating characteristic (ROC) curve was used to evaluate the capability of each blood marker to classify the patients as alive/death or progressive disease/not and the area under the ROC curve (AUC) was calculated. An optimal cut-point value according to the highest difference between true-positive and false-positive predictions was obtained. Overall survival (OS) was defined as time from the date of start concomitant radio-chemotherapy to the date of death from any cause; progression-free survival (PFS) was computed from date of start concomitant radio-chemotherapy therapy to the date of disease progression or death from any cause, whichever came first. PFS and OS were reported as median values with 95% confidence interval (95% CI). Survival curves were estimated using the Kaplan–Meier method (two-sided 95% CIs) and compared with the log-rank test. Estimated HRs with 95% CI were calculated using univariate and multivariate Cox proportional hazard models. Furthermore, we selected a cohort of 31 pts from our population numerically balanced for pre-surgical SII-high and pre-surgical SII-low, to study their immune infiltrate through the archival formalin-fixed paraffin embedded (FFPE) tissue specimens. Thinking about how tumor size can influence the immune component, we considered tumor size on MR imaging at pre-surgery of these 31 patients. Statistical analyses were carried out with Stata software 15.1/SE for Windows, StataCorpLLC, College Station, TX, USA). Time dependent ROC curves were performed using timeROC and survivalROC packages in R software (version 4.2.0). MGMT promoter methylation status was performed on formalin fixed paraffin embedded samples by pyrosequencing technology using a commercially available kit.

### Immunohistochemistry analysis

Surgical specimens embedded in paraffin were sliced with a rotating microtome (Leica Biosystems, Wetzlar, Germany) and 3 µm thick sections were mounted on positive-charged microslides (Thermo Fisher Scientific, Waltman, MA, USA). Immunohistochemistry was performed using the VENTANA Benchmark Ultra (Ventana Medical Systems Inc, Tucson, AZ, USA). The antibodies (Ab) were used for CD3, CD4, CD8, CD20, CD45, CD68, CD163, CD66b and PDL-1. IHC staining was evaluated when tissue cellularity was sufficient. Expression levels were classified according to a Score ranging from 0 to 4 (0 = no expression; 1 = 1–25%; 2 = 26–50%, 3 = 51–74%; 4 = from 75 to 100%). The tissue distribution and intensity of each Ab was recorded to evaluate biomarker positivity in two-tumoral area: Vascular/perivascular (V) and diffuse in tumor parenchyma (D). Percentage of infiltrating immune system cell was calculated by the rate of absolute number of positive stained cells/total number of cells multiplied by 100. The whole process was supervised by two expert pathologists.

## Results

### Inflammation markers

Ninety-five patients were considered in this retrospective study: 61 male (64.2%) and 34 female (35.8%) were included and median age was 61 years (range: 37–77), as shown in Table 1. Sixty-seven patients (72.8%) had a MGMT ≤ 30% and were defined as unmethylated and twenty-five (27.2%) were methylated (MGMT ≥ 30%), while three patients had an unknown status.

**Table 1:** Patients characteristics (n = 95)

Patients characteristics	N (%)
Gender	
Male	61 (64.2)
Female	34 (35.8)
Age at diagnosis	
Median (range)	61 (37–77)
MGMT (2)	
Unmethylated (0–29%)	67 (72.8)
Methylated (≥ 30%)	25 (27.2)
Unknown	3
Surgery	
Gross total removal	35 (37.2)
No gross total removal	59 (62.8)
Unknown	1
PS (ECOG)	
0	35 (36.8)
1	52 (54.8)
2	6 (8.4)
N Temodal cycles	
None	17 (17.9)
1–6	53 (55.8)
> 6	25 (26.3)

In Table 2, descriptive statistics were reported for all the blood markers that increased significantly among pre-surgery and pre-chemotherapy, as well as among pre-chemotherapy and the end of treatment; an exception was made for PLR (*p*-value = 0.570) that had similar values among pre-chemotherapy and the end of treatment. ROC curves were used to select an optimal cut-off value for different blood markers (pre-surgery SII, NLR, PLR, pre-chemo SII, NLR, PLR) related to the OS and PFS. We considered both the inflammatory index at pre-surgery and pre-chemotherapy time. AUC value was discriminant especially for pre-chemo-SII at 480 (Supplementary Table S1).

**Table 2:** Variation of blood markers over time

Blood markers	Pre surgery value (1) Median (iqr range)	Pre chemo value (2) Median (iqr range)	Post treatment value (3) Median (iqr range)	*p*-value from Friedman’s test	Post hoc comparison (Bonferroni’s correction)		
					2 VS. 1	3 VS. 2	3 VS. 1
	N = 95	N = 95	N = 70				
SII	153.7 (126.6–192.0)	604.7 (396.9–1042.1)	576.3 (288.1–1133.3)	< 0.001	< 0.001	< 0.001	1.000
NLR	0.76 (0.65–0.82)	2.5 (1.7–3.8)	3.7 (2.5–5.8)	< 0.001	< 0.001	< 0.001	< 0.001
PLR	20.3 (14.1–30.3)	105.3 (75.0–163.6)	119.9 (85.1–171.8)	< 0.001	< 0.001	0.570	< 0.001

Median OS (mOS) for overall case series was 12.6 months (95% CI 11.3–16.3). Patients aged < 60 years showed better OS in respect to patients ≥ 60 with a median OS of 15.6 months (95% CI 11.3–22.1) vs. 11.9 months (95% CI 9.5–14.9, *p*-value = 0.045); methylated patients had a better mOS (median 19.7 months, 95% CI 11.3–37.4) respect to unmethylated (median 12.2, 95% CI 10.3–15.6, *p*-value = 0.020). *Tumor location, surgery and symptoms were not related to mOS and mPFS* (Supplementary Table 2, 3). Pre-chemotherapy SII < 480 was related to a better OS (median 17.7 months, 95% CI 12.6–22.2 vs. 11.3 months, 95% CI 9.1–12.9, *p*-value = 0.014) (Supplementary Table S2). Pre-chemo NLR and PLR values did not show a prognostic role: patients with NLR < 2.2 had 14.0 months as mOS (95% CI 11.3–20.6), while patients with NLR ≥ 2.2 had a mOS of 11.9 months (95% CI 9.1–15.6, *p*-value = 0.075); patients with PLR < 110 had 15.0 months as mOS (95% CI 11.3–19.8), while patients with PLR ≥ 110 had a mOS of 11.8 months (95%CI 8.0–15.5, *p*-value = 0.306). Patients with pre-surgery PLR values < 31 had a better OS respect to patients ≥ 31 (median 14.9 months, 95% CI 11.8–19.7 vs. 8.9 months, 95% CI 5.5–12.2, *p*-value = 0.010).

Multivariable model (Table 3) was carried out including age and MGMT, because statistically significant in univariable analyses. Younger age, methylation, low value of pre-chemo-SII and pre-surgery-PLR were confirmed as prognostic parameters of OS.

**Table 3:** Univariable and multivariable models for overall survival

			Overall survival			
Characteristics	No of cases	No of events	HR from univariable model (95% CI	*p*-Value	HR from multivariable model (95% CI)	*p*-Value
Age at therapy start						
< 60 years ≥ 60 years	44 51	38 46	1.00 (referent) 1.56 (0.99–2.43)	0.047	1.00 (referent) 1.85 (1.13–3.02)	0.014
MGMT						
Unmethylated (0–29%)	67	61	1.00 (referent)		1.00 (referent)	
Methylated ≥ 30%)	25	20	0.54 (0.32–0.91)	0.023	0.51 (0.30–1.67)	0.014
Prechemo SII						
SII < 480	36	31	1.00 (referent)		1.00 (referent)	
SII ≥ 480	59	53	1.74 (1.11–2.74)	0.015	1.76 (1.10–2.81)	0.018
Presurgery PLR						
PLR < 31	75	65	1.00 (referent)		1.00 (referent)	0.036
PLR < 31	20	19	1.99(1.17–3.40)	0.012	1.83 (1.04–3.20)	

Median PFS (mPFS) for overall series was 6.7 months (95% CI 5.5–8.8). As shown in Table 4, patients with higher MGMT methylation value had a better median survival (12.2 months, 95% CI 9.5–20.4 for methylated patients vs. 5.9 months, 95% CI 4.8–7.4 for unmethylated patients, *p*-value < 0.001). Pre-chemotherapy SII < 480 was associated to a better PFS: 10.7 months (95% CI 8.7–15.4) vs. 5.7 months (95% CI 4.9–6.7, *p*-value = 0.004) with a possible prognostic role. *Among the symptoms at diagnosis, only the motor dysfunction showed a negative impact on PFS (Supplementary Table S3).*

**Table 4:** Univariable and multivariable models for progression-free survival

Characteristics	Progression-free Survival			
	HR from univariable model (95% CI)	p-Value	HR from multivariable model (95% CI)	p-Value
MGMT (30%)				
Unmethylated (0–29%)	1.00 (referent)		1.00 (referent)	
Methylated (≥ 30%)	0.40 (0.25–0.66)	< 0.001	0.40 (0.24–0.66)	< 0.001
SII presurgery value				
SII < 146.6	1.00 (referent)			
SII ≥ 146.6	1.52 (1.01–2.32)	0.048		
Pre chemo SII				
SII < 480	1.00 (referent)		1.00 (referent)	
SII ≥ 480	1.86 (1.20–2.88)	0.005	1.94 (0.92–4.09)	0.080
Pre chemo NLR				
NLR < 2.2	1.00 (referent)		1.00 (referent)	
NLR ≥ 2.2	1.61 (1.06–2.46)	0.025	0.84 (0.43–1.64)	0.614
Pre chemo PLR				
PLR < 110	1.00		1.00	
PLR ≥ 110	1.52 (1.01–2.31)	0.048	1.07 (0.61–1.89)	0.809
Motor dysfunction				
No	1.00			
Yes	1.79 (1.09–2.94)	0.021	2.03 (1.20–3.40)	0.008

Pre-surgery NLR did not show a statistically significant difference with mPFS of 6.7 months (95% CI 5.5–9.4) for patients with NLR < 0.87 and a mPFS of 5.7 months (95% CI 2.7–11.1) for patients with NLR ≥ 0.87. Pre-chemo NLR and PLR values had a significant prognostic role: in particular patients with prechemotherapy NLR value < 2.2 had an higher mPFS (9.2 months, 95% CI 5.7–11.8) respect to patients with NLR value ≥ 2.2 (mPFS 5.9 months, 95% CI 5.1–7.4, *p*-value = 0.023); as well as patients with pre-chemotherapy PLR value < 110 had an higher mPFS (10.5 months, 95% CI 6.5–12.0) respect to patients with PLR value ≥ 110 (mPFS 5.5 months, 95% CI 4.3–6.7, *p*-value = 0.046). In multivariable model (Table 4) not all the variables statistically significant in univariable analysis were included due to collinearity among pre-surgery and pre-chemotherapy SII: only MGMT maintain an independent prognostic role with a lower risk of death for methylated patients (HR:0.40, 95% CI:0.24–0.66); also patients who had motor dysfunction maintain an higher risk of progression, respect to patients who hadn’t it (HR:2.0, 95%CI:1.20–3.40).

### Tissue immune-related markers

**Fig. 1:**
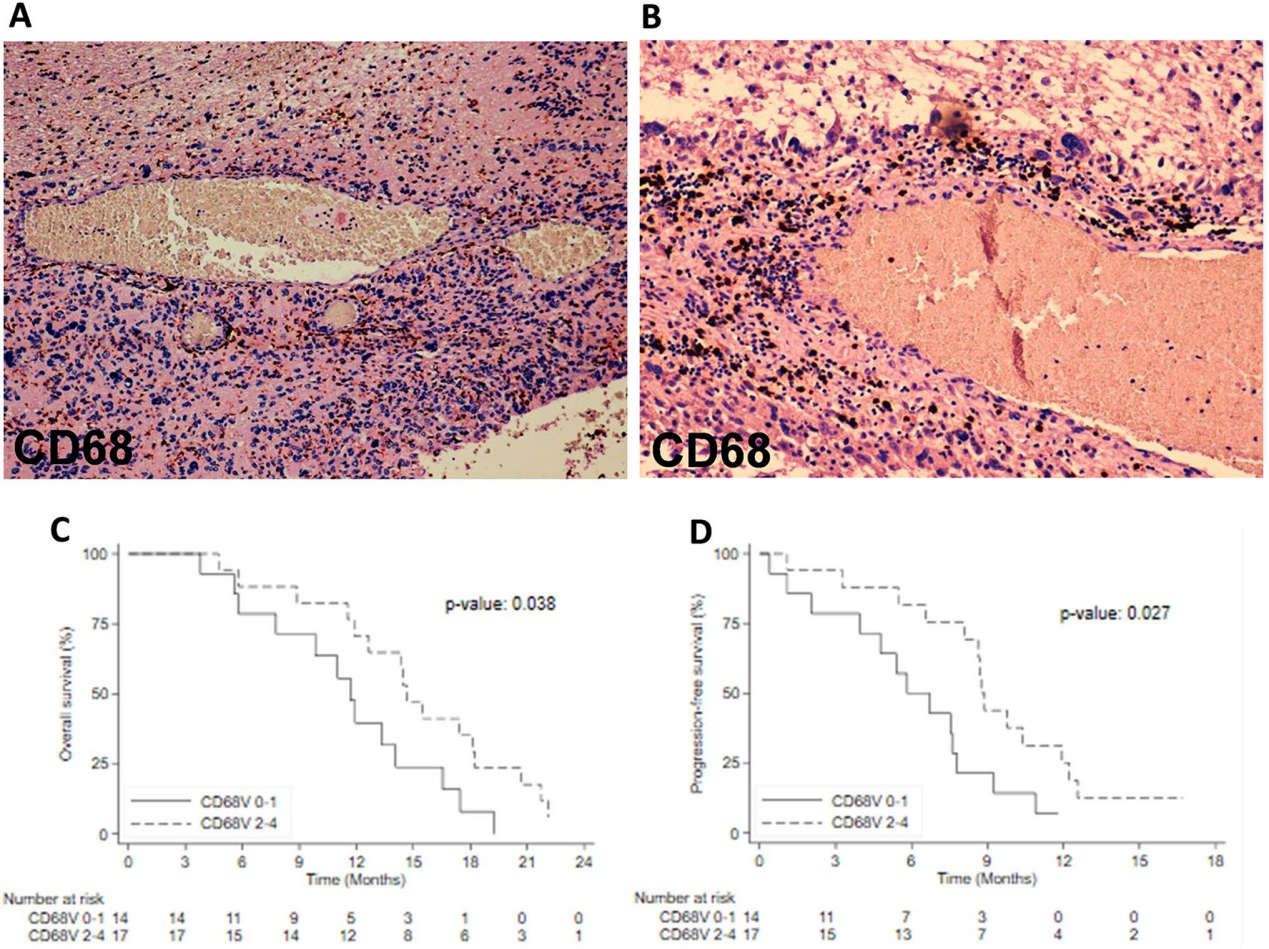

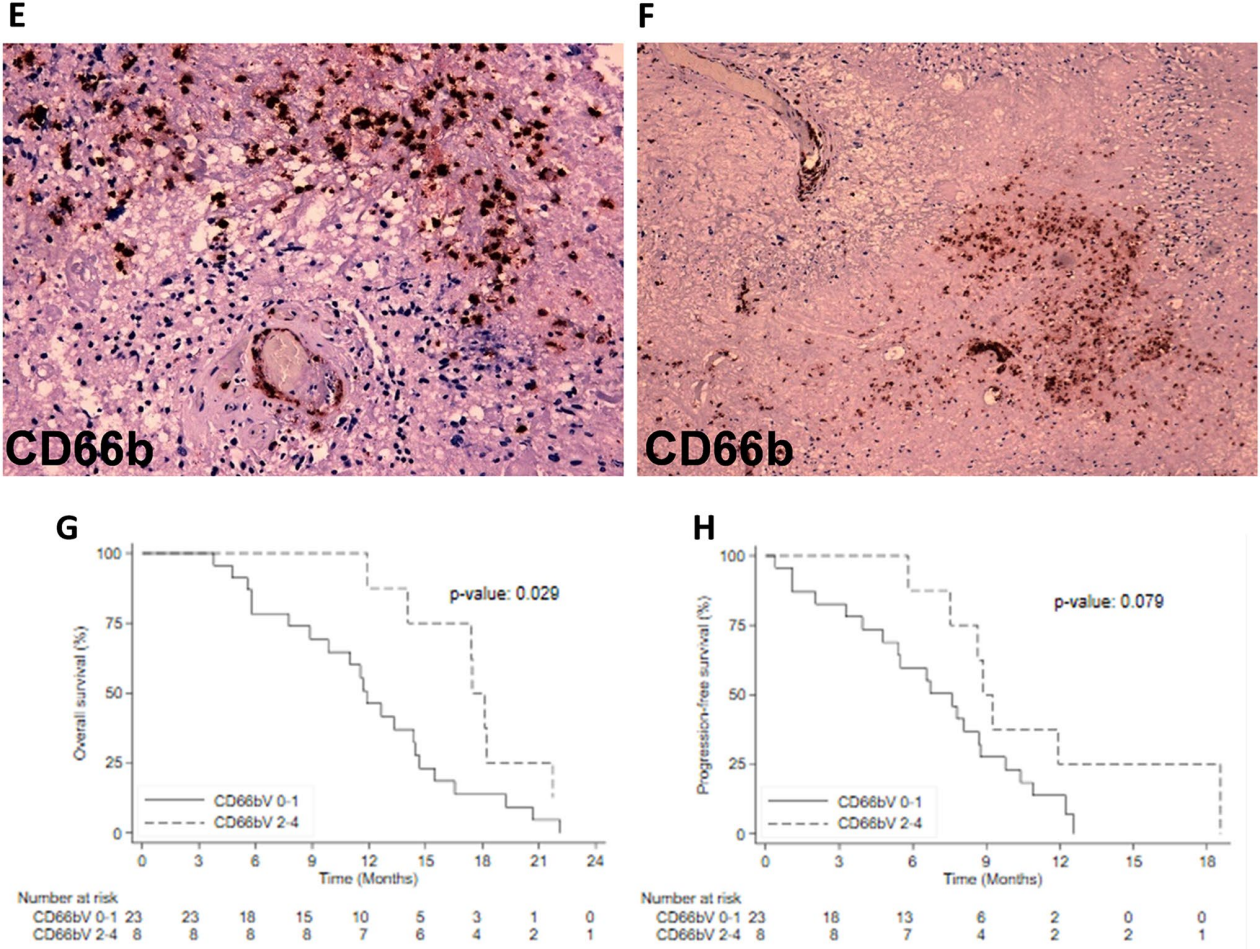
**A**, **B**, **E**, **F** Representative IHC images of tumour-specific characteristics in original GBM for macrophage CD68+(**C**,10×) and neutrophils CD66b+(**F**, 20×, 10×); **C**, **D** Relation between immunomarker of macrophages and overall survival and progression free survival; **G**, **H** relation between immunomarker of neutrophils and overall survival and progression free survival

Evaluation of TILs and TAMs, as previously reported, through their distribution and intensity (score 0–4) as well as their presence in perivascular area (V) and parenchimal tumor (D) was performed on whole slides of 31 resected GBM tissues by IHC. Regarding the immune cells distribution, we considered the CD8/CD163 ratio. Our limit was to work with score (0–4) so we didn’t estimate numerically the details. We observed M2 macrophages CD163+more frequent than lymphocytes according to literature data, in which the necrotic tissue usually is highly infiltrated by macrophages. We have excluded from the analysis PDL-1 and B-lymphocyte subtype marker CD20, because all analyzed tissues were negative for PDL1 and only two were positive for CD20. We focused on the macrophage and monocyte CD68+(Fig. 1A, B) and CD66b+neutrophils (Fig. 1E, F) that were attracted to the tumor by cytokine during inflammation. Of note, when we correlate the expression level with the overall survival, we found that CD68-V and CD66b-V shQueryowed a statistically significant prognostic role reporting a *p*-value of 0.038 and 0.029 respectively (Fig. 1C, D). CD68-V showed a prognostic role for PFS (*p*-value = 0.027, Fig. 1G), while CD66b-V expressions did not (*p*-value = 0.079, Fig. 1H). The expression levels of CD3, CD4, CD8, CD45 and CD163 were not associated with OS and PFS. None of the tissue markers tested correlated with SII pre-surgery as marker of inflammation at diagnosis. Regarding the tumor size in this cohort, we didn’t observe a significant role in mOS (14.1 vs. 11.9mo for size ≤ 50 mm and > 50 mm respectiverly, p. 0.627) and in mPFS (7.8 vs . 8.1mo for size ≤ 50 mm and > 50 mm respectiverly, p. 0.521).

## Discussion

The prognosis of GBM patients is poor even if the patients received Stupp regimen treatment after surgery [1]. In the past studies, MGMT methylation showed an independent prognostic role in the outcome, but it had a role in molecular heterogeneity inside the GBM [20, 32]. In recent years, beyond the molecular factors (i.e., MGMT and IDH1/2), circulating markers of inflammation and immune components within the tumor tissue have been the focus of attention in oncology as potential prognostic factors. Recent studies have considered GBM consisting in immuno-deserted TME, but they did not consider paired blood and tissue samples [33]. Moreover, blood biomarkers represent an attractive candidate due to ease of access, a lower cost and not invasive procedure. Starting from this, we conceived this retrospective work combining both analyses. Our series includes 95 patients affected by GBM, which had undergone neurosurgery and radio-chemotherapy following by chemo (TMZ). We described the OS and PFS according to clinical and molecular data that reflected the literature [34–38]. In particular, the relation between the younger age and the presence of highly MGMT methylation status, had a significant positive prognostic role [3, 4]. In contrast, the tumor location and type of surgery were both not significant. In order to complete clinical data analysis, we had a limit regarding the tumor volume. Considering the patients since 2008, it was not possible to find the digital version of radiological images so we omitted this data. We also evaluated hematologic markers of inflammation and SII at pre-surgery and pre-treatment time-point. From literature data, we know that the presence of inflammatory cells in the TME, could influence the tumor growth and invasion [39]. The first study on inflammatory markers in glioma by Zadora et al., concluded that pre-surgery NLR values were associated with high grade [40]. Yersal et al., performed a study on 80 patients calculating NLR and PLR values pre-chemo and showed a better prognosis for NLR < 4, while for PLR a prognostic role was not found. The authors concluded that these markers alone were not helpful to predict the prognosis in GBM [41]. Liang et al., used SII to perform differential diagnosis between high and low grade glioma; in particular they observed that the extent of neutrophil infiltration was positively related with the grade of the tumor [42]. In contrast to their work, we did not observe the NLR prognostic role both at pre-surgery and pre-chemo time. These two studies had certain limitations: the sample size was not sufficient to reach statistical significance for survival, the unknown MGMT methylation status and incomplete follow-up data. In our series, pre-surgery PLR ≥ 31 had a negative statistically significant impact on OS. This result could be explained thinking about the role of platelets on tumor cells. Indeed, the activation of TGFb and NF-kB pathways, induces an invasive phenotype on cancer cells [43]. As shown in Table 2, our patients had median pre-surgery-NLR value lower than pre-chemo (0.76 vs. 2.5) probably due to the lymphocytes cells which could try to inhibit cancer development before surgery. Kaya et al. in 2017, retrospectively confirmed that OS was significantly correlated with systemic inflammatory response based on NLR count prior to treatment [18]. Our data suggested a different trend of blood markers over time with an important increase in SII, NLR and PLR from pre-surgery to pre-chemo time point, due to the inflammation induced by surgery; later only SII and NLR continued to raise. Applying their variable to our cases, we didn’t observe a significant impact of systemic inflammatory response (*p*-value = 0.406), but considering pre-chemo-SII ≥ 480, we confirmed its poor prognostic role for both OS and PFS which were significantly shorter in these patients (*p*-value = 0.014; *p*-value = 0.004 respectively). Regarding TME in GBM, some studies have already cited the immunosuppressive components, characterized by recruitment of myeloid cells and low anti-tumor lymphocytes, as cause of failure for the immunological therapies [8]. All authors agree on the importance to investigate the immunomodulatory mechanisms involved in GBM TME, to develop future immunotherapeutic strategies [7–9]. In 2020 Koshkaki et al., published the first work on nine GBM demonstrating the different immune cells composition in the tumor core compared to the perivascular area [44]. Indeed, tumor tissues were enriched in immunosuppressive M2 macrophage (CD163+) in both areas, while CD3+T cells were prevalent inside the tumor core, but lower than CD163+ cells, explaining the suppressive effect of TAM on T cells. Finally, they observed more PD1 positive cells in the tumor-core comparing to the perivascular area. Together these findings can partially explain the immunosuppressive role of TAM and the failure of anti-PD1 therapies in GBM. In 31 cases evaluated by IHC, we tested tumor core (D) and perivascular area (V). TIL subpopulation was constituted by a higher number of T-lymphocyte (CD3+) than B-lymphocyte (CD20), and among them CD8+T cells were the most prevalent. Regarding the CD8+/CD163+, the higher presence of M2-macrophage respect to T cells both in D and in V areas reflected the literature data. When we verified the possible prognostic role of each cell component, we found that CD68 expression in V, was associated with a significant positive impact on both OS and PFS. This relation could be partially explained because TAM do not raise only from peripheral blood, but also from resident microglia. TAM is the first help to maintain brain homeostasis but also, they are important to protect brain through their proinflammatory property. Macrophages were distributed with a high density in perivascular area, where they were ready to migrate from blood vessels inside tumor tissue probably giving a positive impact in outcome. A second explanation could be the presence of necrosis, above all in large tumor, because necrotic tissue is highly infiltrated by TAM. The role of neutrophils in glioma is still debated. Fossati G et al. reported that neutrophil inside the tumors are significantly related with glioma grade, and provide a link between inflammation and progression [45]. In contrast, other studies showed that neutrophils can directly exert important antineoplastic activity [46]. Most studies shared that TAM can cooperate with CD66b+activated granulocyte to suppress the immune milieu in the GBM microenvironment. Moreover, we observed high density of CD66b+cells in V probably for growth factors overproducted by tumor cells with their recruitment from blood. We found a significant relation with both OS and PFS for these markers, but in contrast, we weren’t able to obtain reliable data regarding PDL1 may be due to the oldness of the histological material (FFPE tissue blocks with more than 5 years), or for the limited specificity of the utilized anti-PDL1 clone (SP142) [47]. Probably due to the small sample size, in our patient cohort we did not find any significant relation between specific immune infiltrate component and pre-surgery SII obtained from the matched blood samples.

## Conclusions

The main aim of this work was to investigate the role of the immune system in GBM. In particular, the retrospective study confirmed that age remain relevant prognostic factors and, considering that the immune infiltration still ongoing to study, MGMT currently cannot be replaced by other markers. We confirmed the role of inflammation, especially of SII, like NLR and PLR as predictive value of response to Stupp regimen. Despite having a smaller cohort of matched FFPE tissues, regarding the immune infiltrate, we showed a different expression of immune markers with statistically significant value respect to OS and PFS for macrophages and neutrophils in the V area. This difference is a further confirmation that GBM is a heterogeneous disease, in which the tumor core and the peri vascular area are diversely populated by the immune system. The blood could give cost-effective inflammatory markers at diagnosis and predictive of response. In future studies, will be straightly important to have a complete evaluation of GBM from diagnosis to different step of disease. Further studies are need based not only on molecular data, but also on the blood component (NLR, PLR and SII) related to neutrophils/lymphocytes/macrophages density in each area of tumor. This evaluation could be essential to develop therapeutic strategies that aim to hit different components of GBM.

## Supplementary Information

Below is the link to the electronic supplementary material.

Supplementary file1 (PDF 146 KB)

## Data Availability

The datasets generated and analyzed during the current study are available from the corresponding author upon reasonable request.
