# Supplementary material for: Immuno markers in newly diagnosed glioblastoma patients underwent Stupp protocol after neurosurgery: a retrospective series
Source: J Neurooncol. 2023 Aug 16;164(1):55–64. doi: 10.1007/s11060-023-04357-9 (PMC10462527; doi:10.1007/s11060-023-04357-9)
Supplement: Supplementary file 1 — Supplementary file1 (PDF 146 KB) [file 11060_2023_4357_MOESM1_ESM.pdf]

SUPPLEMENTARY INFORMATION

Supplementary Table S1 AUC, Sensitivity and specificity values for different blood markers at 3 and 12 months

Inflammatory index	Outcome	3 months AUC (95%CI)	12 months AUC (95%CI)	Proposed cut off	3 months Sens (%)	3 months Spec (%)	12 months Sens (%)	12 months Spec (%)
**Pre surgery SII**	OS	70.65 (41.67-99.63)	68.67 (57.89-79.45)	146.3	66.7	41.3	76.7	58.0
	PFS	65.19 (52.3-78.07)	65.83 (53.29-78.36)	146.3	81.3	45.6	65.7	60.0
**Pre surgery NLR**	OS	68.84 (47.85-89.83)	56.02 (44.21-67.84)	0.87	33.3	84.8	25.6	92.0
	PFS	54.51 (39.38-69.64)	59.14 (45.93-72.35)	0.87	25.0	82.3	21.4	88.0
**Pre surgery PLR**	OS	51.09 (24.27-77.90)	56.49 (44.61-68.38)	31	0.0	78.3	30.1	88.0
	PFS	50.24 (36.14-64.33)	48.74 (36.29-61.19)	31	18.8	78.5	24.3	88.0
**Pre chemotherapy SII**	OS	80.07 (64.85-95.30)	60.21 (48.66-71.76)	480	100.0	39.1	76.7	52.0
	PFS	57.20 (40.53-73.87)	63.54 (51.03-76.05)	480	68.8	39.2	71.4	64.0
**Pre chemotherapy NLR**	OS	67.75 (54.60-80.91)	56.46 (44.81-68.10)	2.2	100.0	45.7	60.4	50.0
	PFS	55.3 (403.82-69.78)	59.9 (48.16-71.79)	2.2	68.8	46.8	61.4	60.0
**Pre chemotherapy PLR**	OS	73.91 (49.15-98.68)	56.56 (44.76-68.39)	110	66.7	54.4	53.3	62.0
	PFS	59.7 (44.78-74.68)	62.23 (49.66-74.80)	110	62.5	56.9	54.3	76.0

Supplementary Table 2 Univariable analysis for overall survival

Variables	N° pts	N. deaths	Median OS (95%CI)	*p*- value (log-rank test)
All pts	95	84	12.6 (11.3-16.3)	-
Gender				
Male	61	55	12.5 (10.5-15.6)	0.190
Female	34	29	16.3 (9.4-23.1)	
Age at therapy start				
<60 years	44	38	15.6 (11.3-22.1)	0.045
≥60 years	51	46	11.9 (9.5-14.9)	
MGMT (30%)				
Unmethylated (0-29%)	67	61	12.2 (10.3-15.6)	0.020
Methylated (>=30%)	25	20	19.7 (11.3-37.4)	
Surgery				
Gross total removal	35	30	13.6 (10.3-19.7)	0.853
No gross total removal	59	53	12.6 (11.1-16.7)	
PS (ECOG)				
0	35	31	15.9 (11.3-21.3)	0.314
>0	60	53	12.2 (9.5-14.9)	
SII **presurgery** value				
SII <146.6	40	35	16.3 (12.9-19.8)	0.109
SII ≥146.6	55	49	10.6 (9.1-12.2)	
SII **prechemo** value				
SII <480	36	31	17.7 (12.6-22.2)	0.014
SII ≥480	59	53	11.3 (9.1-12.9)	
NLR **presurgery** value				
NLR <0.87	80	70	14.3 (11.8-17.7)	0.542
NLR ≥0.87	15	14	9.7 (8.0-11.8)	
NLR **prechemo** value				
<2.2	42	36	14.0 (11.3-20.6)	0.075
≥2.2	53	48	11.9 (9.1-15.6)	
PLR **presurgery** value				
<31	75	65	14.9 (11.8-19.7)	0.010
≥31	20	19	8.9 (5.5-12.2)	
PLR **prechemo** value				
<110	51	47	15.0 (11.3-19.8)	0.306
≥110	44	37	11.8 (8.0-15.5)	
Combination of NLR and PLR (SIR)				
NLR ≥5 or PLR ≥150	32	28	12.6 (6.5-18.5)	0.406
NLR <5 and PLR <150	63	56	12.9 (11.1-17.7)	

Supplementary Table 3 Univariable analysis for progression-free survival

Variables	N° pts	N. PD	Median PFS (95%CI)	*p*-value (log-rank test)
All pts	95	93	6.7 (5.5-8.8)	-
Gender				
Male	61	60	7.4 (5.5-9.4)	0.261
Female	34	33	5.9 (3.9-11.7)	
Age at therapy start				
<60 years	44	43	8.8 (5.8-11.1)	0.158
≥60 years	51	50	5.7 (4.4-8.7)	
MGMT (30%)				
Unmethylated (0-29%)	67	67	5.9 (4.8-7.4)	<0.001
Methylated (>=30%)	25	23	12.2 (9.5-20.4)	
Surgery				
Gross total removal	35	34	7.3 (5.1-10.5)	0.603
No gross total removal	59	58	6.7 (5.7-9.2)	
PS				
0	35	35	8.7 (5.5-10.7)	0.735
>0	60	58	6.5 (5.2-9.2)	
N Temodal cycles				
None	17	17	3.0 (2.1-4.9)	
1-6	53	52	6.0 (5.4-7.4)	<0.001
>6	25	24	15.4 (11.9-20.4)	
SII **presurgery** value				
SII <146.6	44	43	10.4 (7.5-12.2)	0.045
SII ≥146.6	51	50	5.7 (4.4-6.7)	
SII **prechemo** value				
SII <480	36	35	10.7 (8.7-15.4)	0.004
SII ≥480	59	58	5.7 (4.9-6.7)	
NLR **presurgery** value				
<0.87	80	78	6.7 (5.5-9.4)	0.931
≥0.87	15	15	5.7 (2.7-11.1)	
NLR **prechemo** value				
<2.2	42	40	9.2 (5.7-11.8)	0.023
≥2.2	53	53	5.9 (5.1-7.4)	
PLR **presurgery** value				
< 31	75	73	8.1 (5.8-10.4)	0.198
≥ 31	20	20	5.1 (3.4-8.8)	
PLR **prechemo** value				
<110	51	51	10.5 (6.5-12.0)	0.046
≥110	44	42	5.5 (4.3-6.7)	
Systemic inflammatory response from prechemo values (**prechemo**)				
NLR ≥5 or PLR ≥150	32	2	5.5 (3.4-6.7)	0.064
NLR <5 and PLR <150	63	1	8.8 (6.1-10.9)	
